# Streptococcal dTDP‐L‐rhamnose biosynthesis enzymes: functional characterization and lead compound identification

**DOI:** 10.1111/mmi.14197

**Published:** 2019-01-31

**Authors:** Samantha L. van der Beek, Azul Zorzoli, Ebru Çanak, Robert N. Chapman, Kieron Lucas, Benjamin H. Meyer, Dimitrios Evangelopoulos, Luiz Pedro S. de Carvalho, Geert‐Jan Boons, Helge C. Dorfmueller, Nina M. van Sorge

**Affiliations:** ^1^ Department of Medical Microbiology University Medical Center Utrecht, Utrecht University Heidelberglaan 100 3584 CX Utrecht The Netherlands; ^2^ Division of Molecular Microbiology, School of Life Sciences University of Dundee Dow Street DD1 5EH Dundee UK; ^3^ Department of Chemistry, Complex Carbohydrate Research Center The University of Georgia 315 Riverbend Road Athens USA; ^4^ Mycobacterial Metabolism and Antibiotic Research Laboratory The Francis Crick Institute London UK; ^5^ Department of Medical Chemistry and Chemical Biology, Utrecht Institute Pharmaceutical Science University Utrecht Utrecht 3508 TB The Netherlands

## Abstract

Biosynthesis of the nucleotide sugar precursor dTDP‐L‐rhamnose is critical for the viability and virulence of many human pathogenic bacteria, including *Streptococcus pyogenes* (Group A *Streptococcus*; GAS), *Streptococcus mutans* and *Mycobacterium tuberculosis*. Streptococcal pathogens require dTDP‐L‐rhamnose for the production of structurally similar rhamnose polysaccharides in their cell wall. Via heterologous expression in *S. mutans*, we confirmed that GAS RmlB and RmlC are critical for dTDP‐L‐rhamnose biosynthesis through their action as dTDP‐glucose‐4,6‐dehydratase and dTDP‐4‐keto‐6‐deoxyglucose‐3,5‐epimerase enzymes respectively. Complementation with GAS RmlB and RmlC containing specific point mutations corroborated the conservation of previous identified catalytic residues. Bio‐layer interferometry was used to identify and confirm inhibitory lead compounds that bind to GAS dTDP‐rhamnose biosynthesis enzymes RmlB, RmlC and GacA. One of the identified compounds, Ri03, inhibited growth of GAS, other rhamnose‐dependent streptococcal pathogens as well as *M. tuberculosis* with an IC_50_ of 120–410 µM. Importantly, we confirmed that Ri03 inhibited dTDP‐L‐rhamnose formation in a concentration‐dependent manner through a biochemical assay with recombinant rhamnose biosynthesis enzymes. We therefore conclude that inhibitors of dTDP‐L‐rhamnose biosynthesis, such as Ri03, affect streptococcal and mycobacterial viability and can serve as lead compounds for the development of a new class of antibiotics that targets dTDP‐rhamnose biosynthesis in pathogenic bacteria.

## Introduction

**Figure 1:**
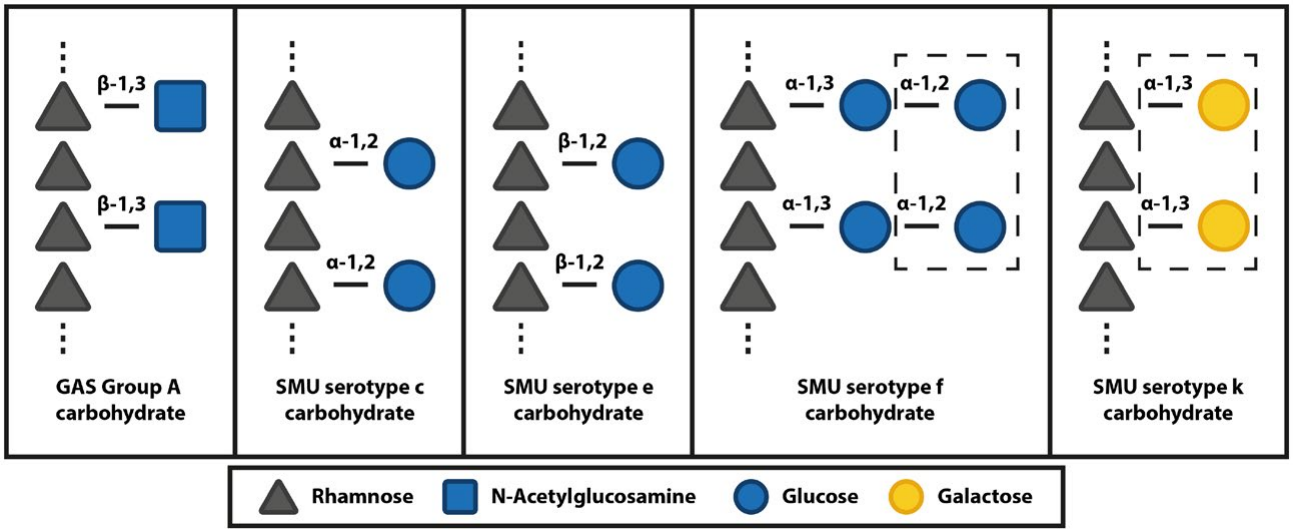
Schematic overview of streptococcal rhamnose polysaccharide structures. Schematic representation of the chemical composition of the GAC from GAS and the serotype‐specific (c, e, f and k) carbohydrates from *S. mutans* (SMU). All carbohydrates share an α‐1,2/α‐1,3 linked polyrhamnose backbone. Sugar residues in dashed boxes were recently identified by St Michael *et al*. (2017).

The typical cell wall architecture of a Gram‐positive bacterium consists of a thick peptidoglycan layer that is decorated with wall teichoic acids, proteins and capsular polysaccharides. However, certain lactic acid bacteria, particularly streptococcal species, lack the expression of wall teichoic acids and instead express rhamnose cell wall polysaccharides, which are covalently anchored to peptidoglycan (Mistou *et al.*, 2016). Rhamnose cell wall polysaccharides encompass about 40–60% of the cell wall mass and are considered to be functional homologs of wall teichoic acids (Sutcliffe *et al.*, 2008; Caliot *et al.*, 2012). Examples of rhamnose cell wall polysaccharides are the group A carbohydrate (GAC) of Group A *Streptococcus *(GAS; *Streptococcus pyogenes*) and the serotype‐determining polysaccharides, referred to as rhamnose‐glucose polysaccharides (RGP), of *Streptococcus mutans*. The GAC and RGP share structural similarities; both consist of an α‐1,2‐/α‐1,3‐linked polyrhamnose backbone with alternating *N*‐acetylglucosamine side chains for GAS and glucose or galactose side chains for *S. mutans* (Heymann *et al.*, 1964; Linzer *et al.*, 1985; Huang *et al.*, 1986; Pritchard *et al.*, 1986; St Michael *et al.*, 2017) (Fig. 1). For *S. mutans*, the type of side chain as well as their linkage to the rhamnan backbone determines the *S. mutans *serotype (Nakano and Ooshima, 2009; St Michael *et al.*, 2017) (Fig. 1). In contrast, all GAS serotypes express a structurally invariant GAC (Lancefield, 1933). Similar to classical wall teichoic acids, rhamnose cell wall polysaccharides are critical for maintaining cell shape, bacterial physiology and virulence, but in‐depth knowledge of their biosynthesis or host interactions at a molecular level is limited (van Sorge *et al.*, 2014; van der Beek *et al.*, 2015; Kovacs *et al.*, 2017; Zhu *et al*., 2017). A better understanding of these mechanisms could aid the development of new classes of antibiotics, antibiotic adjuvants or vaccines (Sabharwal *et al.*, 2006; van Sorge *et al.*, 2014; St Michael *et al.*, 2017).

L‐Rhamnose is the main building block for both the GAC and RGP. The biosynthesis pathway of the nucleotide precursor, dTDP‐L‐rhamnose, is highly conserved among both Gram‐positive and Gram‐negative bacteria (Giraud and Naismith, 2000; Mistou *et al.*, 2016) and critical or even essential for viability or virulence of a wide range of human pathogens including GAS (Le Breton *et al.*, 2013; 2015), Group B *Streptococcus* (GBS) (Caliot *et al.*, 2012; Hooven *et al.*, 2016), some serotypes of *Streptococcus pneumoniae* (Kim *et al.*, 1999; Magee and Yother, 2001), *S. mutans* (Tsuda *et al.*, 2000; Engels‐Deutsch *et al.*, 2003; Kovacs *et al.*, 2017; Shields *et al*., 2018), *Enterococcus faecalis *(Xu *et al.*, 2000; Rigottier‐Gois *et al.*, 2015), *Mycobacterium spp.* (Ma *et al.*, 2002; Li *et al.*, 2006), *Pseudomonas spp.* (Engels *et al.*, 1985) and *Salmonella enterica* serovar *Typhimurium* (Joiner, 1988). Consequently, this pathway is considered to be an interesting drug target, especially since dTDP‐L‐rhamnose is not produced or used by humans (Adibekian *et al.*, 2011).

dTDP‐L‐rhamnose is produced through a four‐step enzymatic pathway catalyzed by the enzymes RmlABCD. In the first step of the pathway, RmlA, a glucose‐1‐phosphate thymidyltransferase, converts glucose‐1‐phosphate into dTDP‐glucose (Blankenfeldt *et al.*, 2000), which is subsequently oxidized and dehydrated to form dTDP‐4‐keto‐6‐deoxy‐D‐glucose by the dTDP‐D‐glucose 4,6‐dehydratase RmlB (Beis *et al.*, 2003). RmlC catalyzes an unusual double epimerization reaction (Giraud *et al.*, 2000; Dong *et al.*, 2003b; 2007), the product of which is finally reduced by RmlD, a dTDP‐4‐dehydrorhamnosereductase, to form dTDP‐L‐rhamnose (Blankenfeldt *et al.*, 2002; van der Beek *et al.*, 2015). The mechanisms of action as well as structural characteristics of the RmlABCD enzymes have been studied extensively (Blankenfeldt *et al.*, 2000; Giraud and Naismith, 2000; Allard *et al.*, 2001; Beis *et al.*, 2003; Dong *et al.*, 2003a; 2003b; Kantardjieff *et al.*, 2004; van der Beek *et al.*, 2015). Indeed, crystal structures are available for all four Rml enzymes from different Gram‐positive and Gram‐negative bacterial species and show high structural conservation. This structural and functional information has enabled the development of different screening methods to discover inhibitors against RmlBCD, yielding compounds that can inhibit dTDP‐L‐rhamnose biosynthesis in the low micromolar range (1–100 µM) in biochemical assay and inhibit growth of *Mycobacterium* *in vitro *(Ma *et al.*, 2001; Babaoglu *et al.*, 2003; Sivendran *et al.*, 2010; Wang *et al.*, 2011; Ren *et al.*, 2015). Many of the identified compounds have limited solubility in aqueous solutions. Therefore, further biochemical and structural information on this pathway in additional species is important to optimize lead compounds for improved affinity and specificity through rational design.

Recently, we characterized GAS GacA as a RmlD homolog catalyzing the last step in dTDP‐L‐rhamnose biosynthesis (van der Beek *et al.*, 2015). Surprisingly, this GAS RmlD enzyme was confirmed to be a monomer as opposed to previously characterized RmlD dimers. Additional bioinformatics analysis of 213 RmlD homologs revealed that the majority of RmlD enzymes are predicted to be monomeric, while only a subclass of RmlD enzymes in Gram‐negatives form metal‐dependent homodimers (Blankenfeldt *et al.*, 2002). In this study, we extended our work to study GAS RmlB and RmlC on a functional level through heterologous expression in *S. mutans* and subsequent analysis of growth, morphology and cell wall composition. In addition, we report the identification of small chemical fragments that bind these enzymes and inhibit GAS growth with IC_50_s ranging from 100 to 300 µM (Ri01, Ri02, Ri03 and Ri06) to 2.7 mM (Ri08). For one compound, Ri03, we confirmed inhibition of dTDP‐L‐rhamnose in a biochemical assay. Furthermore, Ri03 could inhibit growth of *S. mutans, S. equi* subsp. *zooepidemicus* (Group C *Streptococcus*, GCS) and *Mycobacterium tuberculosis* with similar efficacy. These results demonstrate that rhamnose biosynthesis inhibitors can directly interfere with bacterial viability and could form a new class of antibiotics targeting nucleotide sugar production.

## Results

### GAS RmlB and RmlC functionally replace *S. mutans* homologs

**Figure 2:**
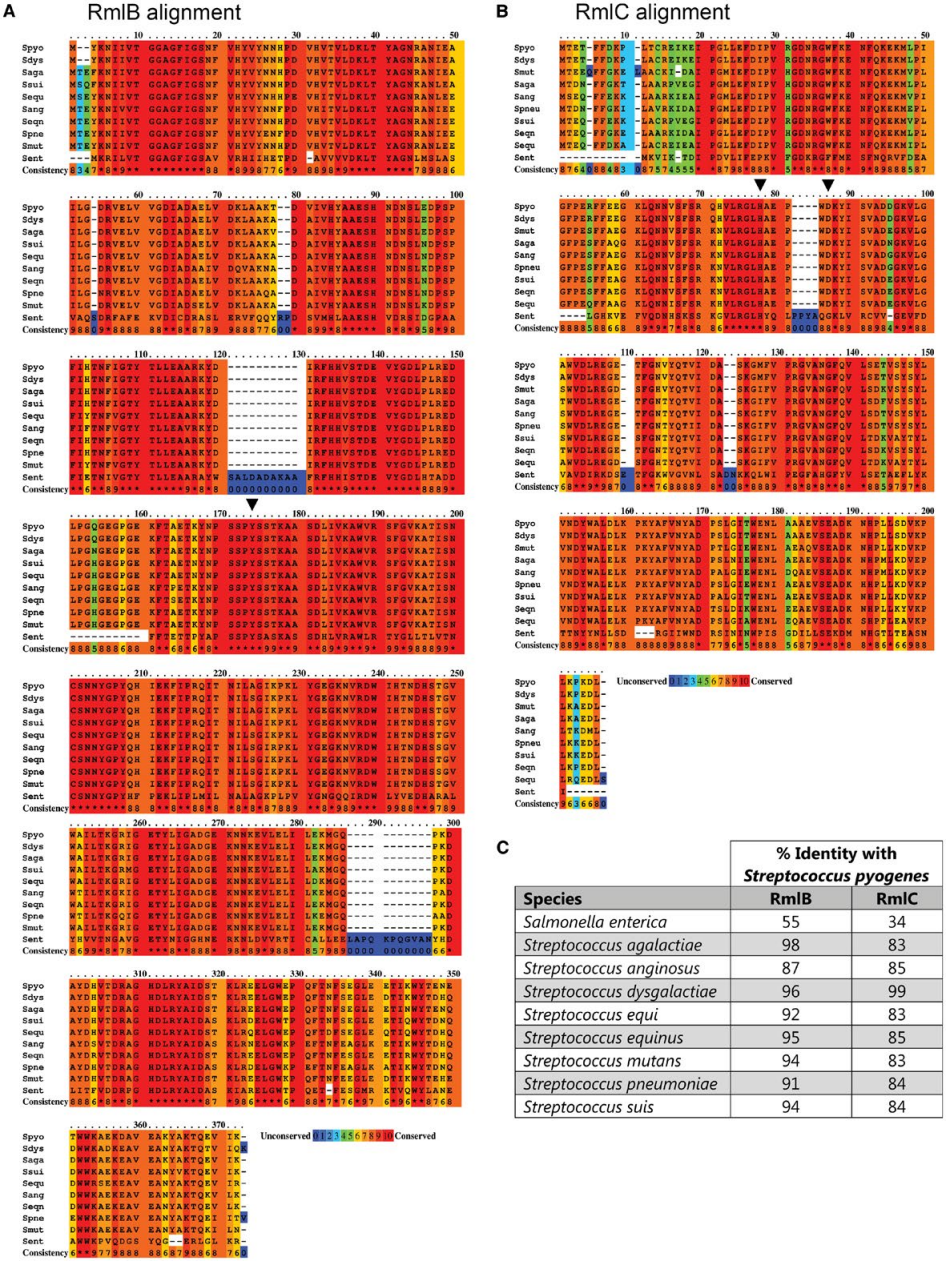
Protein sequence alignment and identity matrix of RmlB and RmlC homologs. Color‐coded representation of amino acid conservation for (A) GAS RmlB and (B) GAS RmlC to S.* enterica* and different streptococcal species. The amino acid conservation is scored from 0 to 10, with 0 (color blue) assigned to the least conserved residue and 10 (color red) to the most conserved residue. Critical enzymatic residues for RmlB (Y159) and RmlC (H76 and K82) are indicated with an inverted triangle. *S. pyogenes *(Spyo); *Salmonella enterica*; (Sent); *S. agalactiae *(Saga); *Streptococcus anginosus *(Sang); *Streptococcus dysgalactiae* (Sdys); *Streptococcus equi *(Sequ); *Streptococcus equinus *(Seqn); *S. mutans* (Smut); *S. pneumoniae *(Spneu); *Streptococcus suis *(Ssui)*.* Protein accession numbers are described in the supplementary data (Table S2). C. Percentage identity matrix of RmlB and RmlC homologs.

As an extension of our previous work on GAS GacA (van der Beek *et al.*, 2015), we sought to characterize GAS RmlB (GAS5448_RS05645) and RmlC (GAS5448_RS05650), the putative dTDP‐glucose‐4,6‐dehydratase and dTDP‐4‐keto‐6‐deoxyglucose‐3,5‐epimerase respectively. GAS *rmlB* and *rmlC* are clustered in an operon together with *rmlA* in the order *rmlACB*. Structural and biochemical analysis of RmlB and RmlC from *Streptococcus suis*, *S. enterica* and *M. tuberculosis* revealed that both enzymes are functional homodimers in these organisms (Giraud *et al.*, 2000; Allard *et al.*, 2001; Beis *et al.*, 2003; Dong *et al.*, 2003b; Kantardjieff *et al.*, 2004). Protein sequence alignment of RmlB and RmlC homologs from several streptococcal species and *S. enterica* displayed high homology (Fig. 2, Table S2). Importantly, all catalytic residues in RmlB and RmlC are conserved (Fig. 2, Table S2).

Most genes directly or indirectly involved in the GAC biosynthesis pathway are essential for GAS viability, including all four dTDP‐L‐rhamnose biosynthesis genes *rmlABC* and *gacA *(Le Breton *et al.*, 2013; 2015; van der Beek *et al.*, 2015; Zhu *et al*., 2017). In contrast and for unknown reasons, the dTDP‐L‐rhamnose biosynthesis genes are not essential in *S. mutans* under noncompetitive conditions. However, gene deletions result in strongly attenuated growth and severe morphological defects (Tsukioka *et al.*, 1997; van der Beek *et al.*, 2015). GAS RmlB and RmlC proteins share 94% and 83% protein sequence identity with their *S. mutans *homologs respectively. In addition, the organization of these genes is identical in both species with the exception of a hypothetical protein (174 nucleotides, 57 amino acids) encoded between RmlC and RmlB.

**Figure 3:**
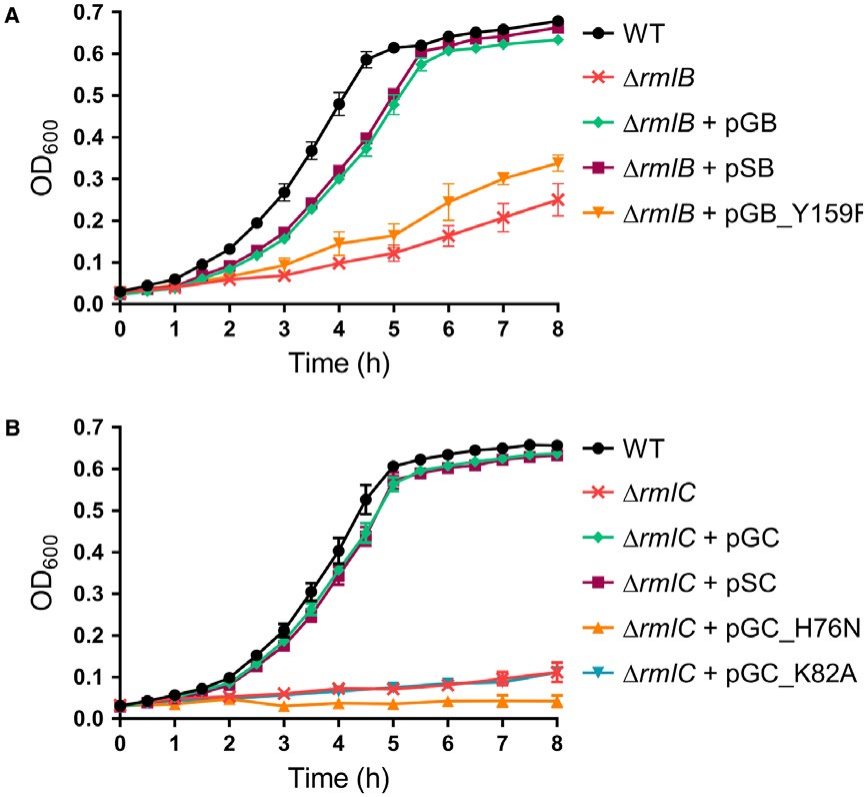
Heterologous expression of GAS RmlB and RmlC and catalytically inactive enzymes in *S. mutans*: growth curves. Growth curves of *S. mutans* (A) *rmlB* and (B) *rmlC* mutant sets: wild type (WT), Δ*rmlB*, Δ*rmlC*, Δ*rmlB* + pSB, Δ*rmlB* + pGB(_Y159F), Δ*rmlC* + pSC and Δ*rmlC* + pGC(_H76N/K82A). Growth curves represent mean ± standard error of mean (SEM) of at least three biological repeats.

**Figure 4:**
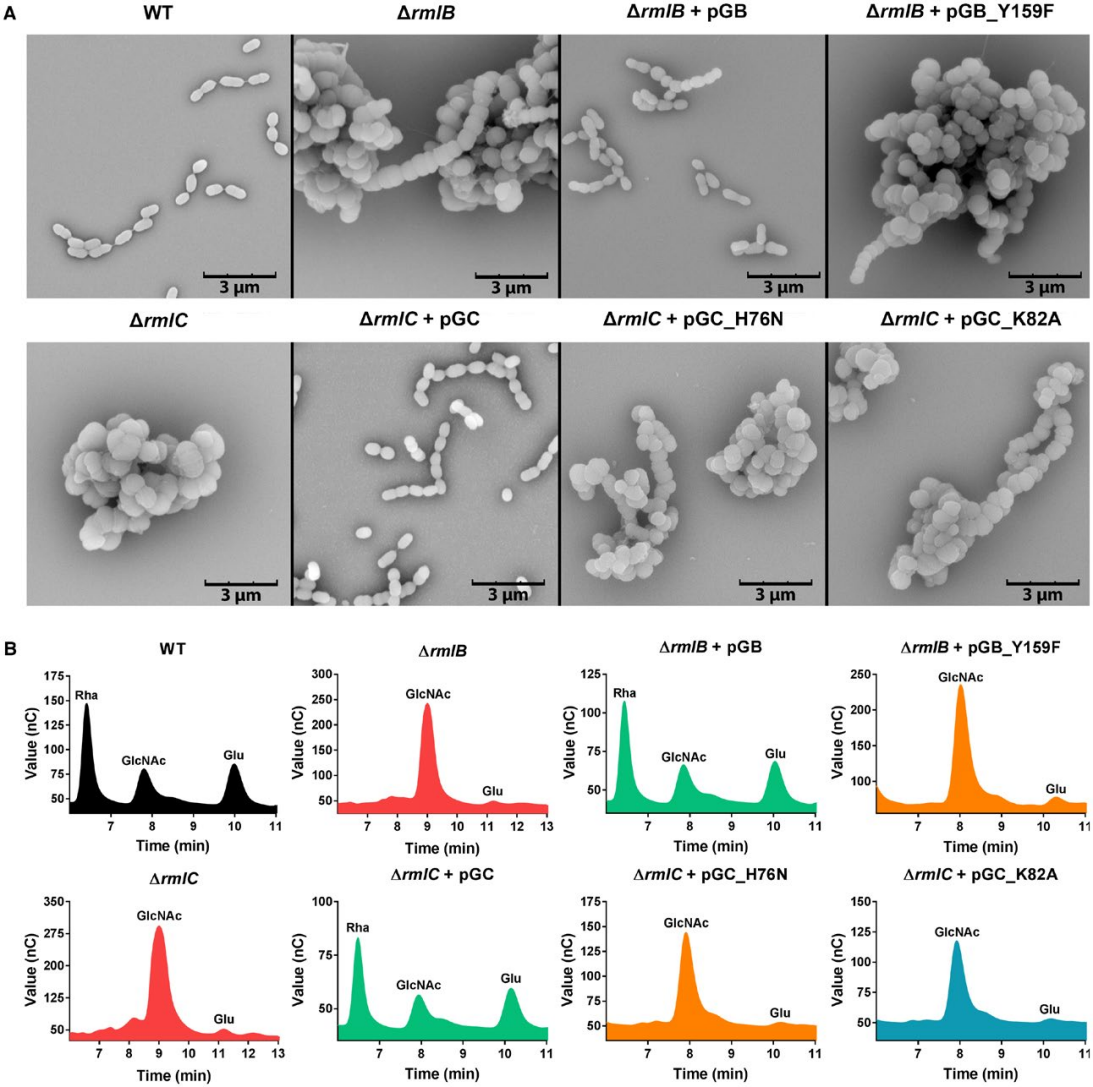
Heterologous expression of GAS RmlB and RmlC and catalytically inactive enzymes in *S. mutans*: morphology and cell wall content. A. Representative scanning electron microscopy images and (B) cell wall carbohydrate composition analysis of *S. mutans *wild type (WT), Δ*rmlB*, Δ*rmlC*, Δ*rmlB* + pGB(_Y159F), and Δ*rmlC* + pGC(_H76N/K82A). Rha, rhamnose; GlcNAc, N‐acetylglucosamine; Glu, glucose.

We took advantage of the nonessential nature of the rhamnose biosynthesis genes in *S. mutans* to confirm the function of GAS RmlB and RmlC in dTDP‐L‐rhamnose biosynthesis. To this end, GAS RmlB and GAS RmlC‐encoding genes were heterologously expressed in *S. mutans* strains lacking *rmlB* or *rmlC* respectively. Deletion of *S. mutans rmlB* (SMU Δ*rmlB*) or *rmlC* (SMU Δ*rmlC*) by replacement with an erythromycin resistance cassette severely attenuated bacterial growth compared to the wild‐type strain (WT) (Fig. 3). This phenotype is characteristic for a rhamnose‐deficient *S. mutans *strain and in line with our previously constructed *S. mutans rmlD *deletion strain (van der Beek *et al.*, 2015). Morphological analysis of *rmlB* and *rmlC* mutant bacteria by scanning electron microscopy revealed swelling and clumping of bacteria as a result of misplaced septa resulting in division errors and multidirectional growth (Fig. 4A). Subsequent analysis of the cell wall carbohydrate composition by HPLC/MS confirmed that SMUΔ*rmlB* and SMUΔ*rmlC* lacked rhamnose in their cell walls, which concordantly resulted in the loss of the glucose side chains (Fig. 4B). Introduction of either homologous *S. mutans rmlB* or heterologous GAS *rmlB* on an expression plasmid in the corresponding SMU Δ*rmlB* mutant restored rhamnose incorporation in the cell wall (Fig. 4B) as well as the defective morphological phenotype and growth (Figs 3 and 4A). Initially, we were unable to complement SMUΔ*rmlC* with *rmlC* from *S. mutans*, whereas heterologous GAS *rmlC* could restore growth, morphology and rhamnose production of the SMUΔ*rmlC* mutant (Figs 3B and 4). Upon reexamination of the UA159 genome, a *S. mutans *serotype c strain that we used as a reference genome for *S. mutans *Xc, we located an alternative ATG‐start site 135 bp upstream of the annotated *rmlC* gene, which is in line with the annotation of *rmlC* in GAS. Importantly, available structural information indicates that the first 45 amino acids are part of the RmlC dimerization interface, forming the extension of the beta‐sheet with two additional beta‐strands, which are required for nucleotide binding (Christendat *et al.*, 2000; Giraud and Naismith, 2000). In agreement with structural and genetic predictions, complementation of SMU Δ*rmlC* with the extended PCR product complemented all observed defects (Figs 3B and 4), indicating that the extended genomic PCR product encodes a functional RmlC enzyme (198 amino acids), which is similar to the size of GAS and *S. suis* RmlC (197 amino acids).

### Catalytic residues of GAS RmlB and RmlC are conserved

Based on the conformation of catalytic residues in *S. suis *RmlB and RmlC (Allard *et al.*, 2001; Dong *et al.*, 2003b) and protein sequence alignment with the GAS RmlB and RmlC homologs (Fig. 2), we set out to functionally validate predicted catalytic residues of GAS RmlB (Y159) and RmlC (H76 and K82). Point mutations were individually introduced in GAS RmlB (Y159F) and RmlC (H76N and K82A) and overexpression vectors carrying these mutant genes were expressed in SMU Δ*rmlB* and SMUΔ*rmlC* respectively. The residues that were mutated are all important for enzymatic activity, since the mutated genes were unable to complement the characteristic rhamnose‐depleted phenotype consisting of growth retardation and an aberrant morphology (Figs 3 and 4A). Correspondingly, our cell wall composition analysis revealed that the *S. mutans *strains carrying these three mutated constructs completely lacked incorporation of rhamnose in their cell wall (Fig. 4B). The functional characterization and confirmation of catalytic residues of streptococcal RmlB and RmlC is important for identification, characterization and optimization of potential dTDP‐L‐rhamnose biosynthesis inhibitors through a rational design approach.

### Inhibitor screen against GAS Rml proteins and hit confirmation

**Figure 5:**
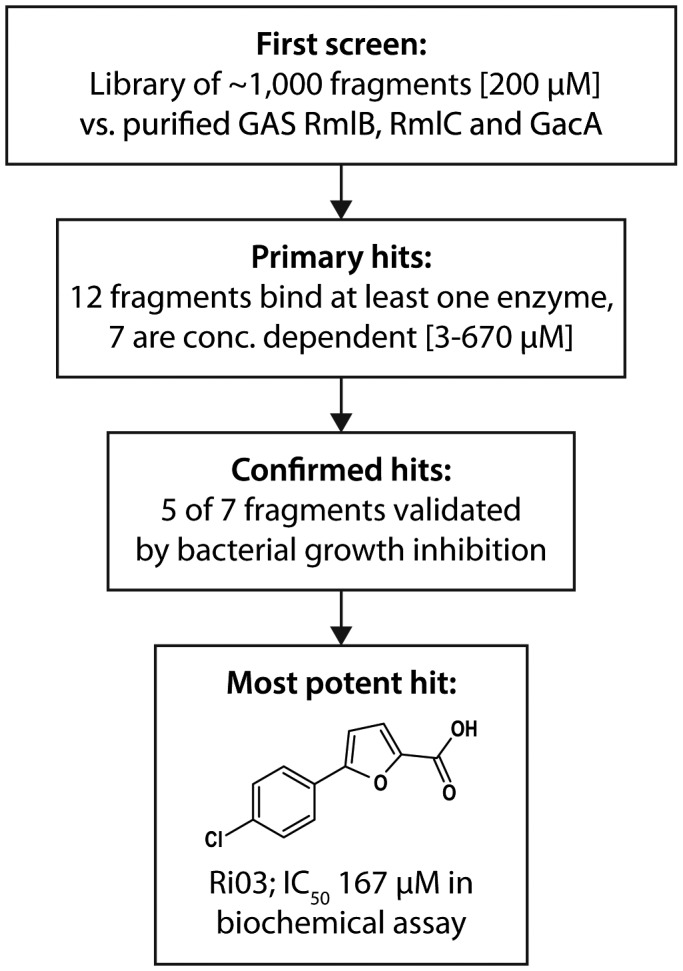
Screening and fragment validation flowchart.

Given the importance of the dTDP‐L‐rhamnose biosynthesis pathway for viability or virulence of many human pathogens, we aimed to identify chemical scaffolds that could act as starting points for future optimization and drug development. In addition, such compounds could also find applications in chemical biological studies aimed at understanding the biosynthesis of dTDP‐L‐rhamnose, particularly when a genetic approach is not possible or desired. Therefore, we conducted a bio‐layer interferometry (BLI) inhibitor screen against the commercially available Maybridge Library using the three recombinant GAS dTDP‐rhamnose biosynthesis enzymes RmlB, RmlC and GacA (Fig. S1A). The advantage of this approach is that it precludes the use of expensive or commercially unavailable enzyme substrates. Using a commercially available library of ~1,000 chemical fragments, our initial screen identified 12 hits (Figs 5, S1B‐D) of which seven fragments were found to specifically interact with GAS RmlB/RmlC/GacA as confirmed by BLI (Figs 5, S2). In addition, we confirmed the identified hits in dilution series for their binding affinity to these three enzymes (Fig. S2). Importantly, this method allowed us to estimate enzyme specificity by comparing the data obtained from one enzyme to the other two. Using this strategy, we identified chemical scaffolds with specificity for one enzyme, but also several fragments that bound to more than one enzyme of the dTDP‐rhamnose biosynthesis pathway (Figs 5, S2, and Table 1).

**Table 1:** Overview of identified compounds and IC_50_ for GAS growth inhibition

Inhibitor	Compound	Binding target	IC_50_ (mM) GAS
Ri01	N‐(1‐Naphthyl)ethylenediamine dihydrochloride	RmlB, RmlC, GacA	0.27
Ri02	2,5‐Difluorophenylhydrazine	RmlB, GacA	0.27
Ri03	5‐(4‐chlorophenyl)‐2‐furoic acid	RmlB, RmlC, GacA	0.12
Ri06	4,4'‐Thiodiphenol	RmlB, GacA	0.11
Ri08	2‐(2,5‐Dimethyl‐1H‐pyrrol‐1‐yl) benzoic acid	RmlB, RmlC	2.67

### Bactericidal activity of identified fragments

**Figure 6:**
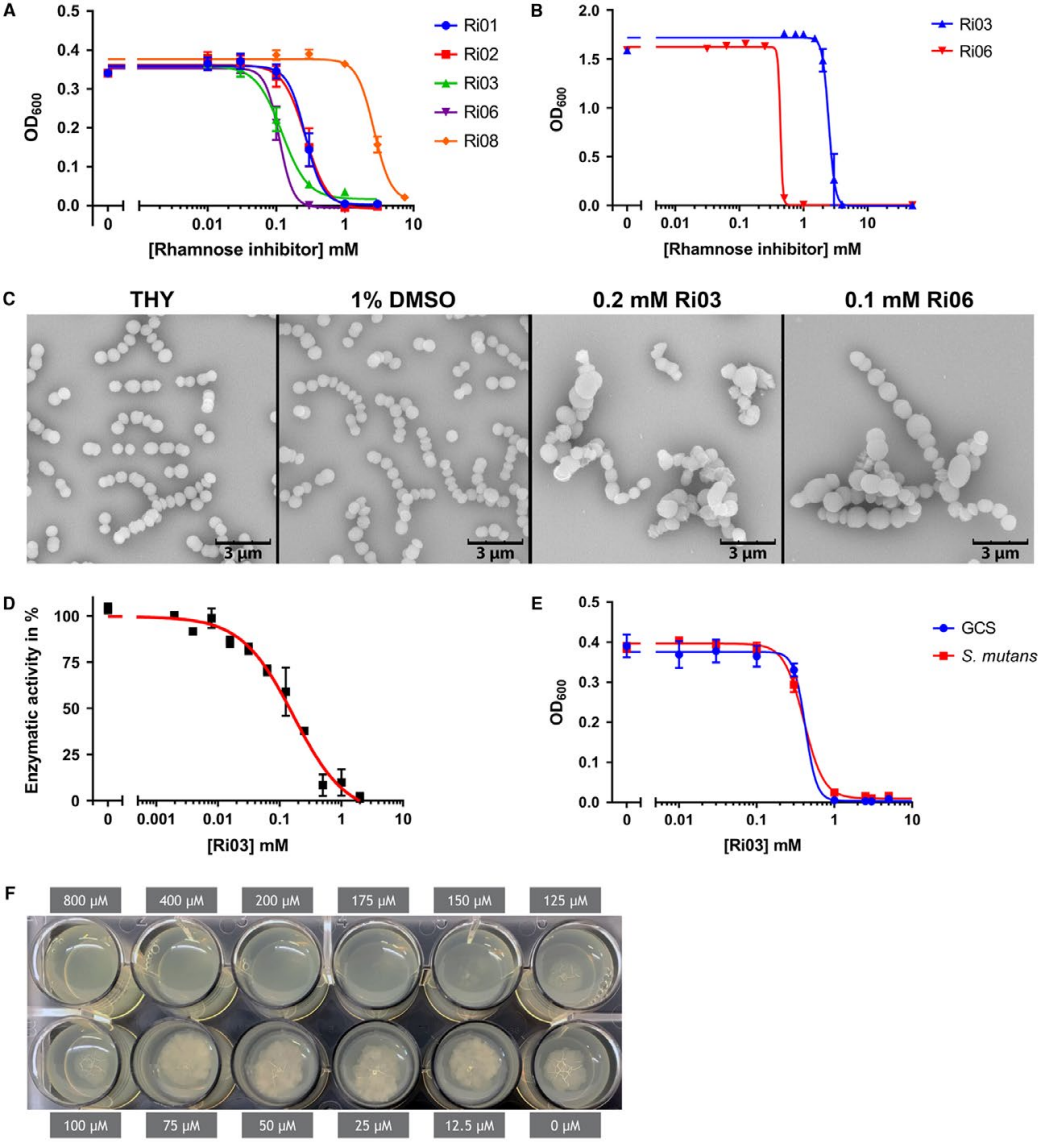
Fragment Ri03 inhibits dTDP‐L‐rhamnose biosynthesis *in vitro* and growth of streptococci and *M. tuberculosis* *in vivo*. A. Dose–response curves for growth inhibition of GAS with various concentrations of Ri01, Ri02, Ri03, Ri06 or Ri08. IC_50_ values range from 110 µM to 2.67 mM. B. Dose–response curves for growth inhibition of rhamnose‐deficient *S. aureus* with various concentrations of Ri03 and Ri06. IC_50_ values range are 2.48 mM and 440 µM respectively. C. Representative scanning electron microscopy images of GAS after 16 h incubation in growth medium (THY), or THY with the addition of 1% DMSO, 0.2 mM Ri03 or 0.1 mM Ri06. D. Dose‐dependent inhibition of dTDP‐L‐rhamnose biosynthesis using recombinant RmlB, C and GacA in a biochemical assay. The assay was initiated by the addition of 400 µM dTDP‐D‐glucose and conversion was measured through oxidation of NADPH to NADP+ at 340 nm. Background absorbance was subtracted and IC_50_ value was determined using the nonlinear fit model E. Dose–response curves for growth inhibition of *S. equi *subsp. *zooepidemicus* (Group C *Streptococcus*; GCS) and *S. mutans* with various concentrations of Ri03. IC_50_ values are 420 µM and 410 µM respectively. F. Ri03 prevents growth of *M. tuberculosis* with a minimal inhibitory concentration of 175 µM using an agar‐based spot culture growth inhibition assay. Dose–response curves represent mean ± SEM of at least three biological experiments performed in duplicate, except for *M. tuberculosis* MIC, which was performed twice.

Since dTDP‐rhamnose is an essential nucleotide sugar in GAS (Le Breton *et al.*, 2013; 2015), we assessed whether these fragments could affect GAS growth. All fragments identified in this study were water insoluble and were therefore dissolved in DMSO, except Ri07, which was already liquid at room temperature. GAS is tolerant to DMSO concentrations of 2%, therefore this was the maximum concentration used in bacterial assays. Even in DMSO, Ri04 and Ri07 were highly insoluble in bacterial culture medium and could therefore not be tested for inhibition of bacterial growth. The remaining five fragments were soluble at the highest concentration tested and were able to inhibit growth of GAS with IC_50 _values ranging from 110 µM to 2.67 mM (Fig. 6A, Table 1). Since Ri03 and Ri06 were most active against GAS, we continued with these two fragments. In accordance with growth inhibition, GAS morphology was also severely affected upon exposure to either 200 µM Ri03 or 100 µM Ri06 (Fig. 6C). Especially for Ri03, streptococci were swollen and chains were longer, a phenotype reminiscent of an inducible *gacA* GAS knockout strain (van der Beek *et al.*, 2015).

The fragments that were identified here may not be completely specific for inhibition of dTDP‐L‐rhamnose since they generally have lower affinity. To assess potential off‐target effects, we incubated Ri03 and Ri06 with *Staphylococcus aureus* USA300 NRS384, a pathogenic methicillin‐resistant bacterium that lacks expression of rhamnose. Ri03 and Ri06 killed *S. aureus *with an IC_50_ ~ 2.48 mM and 440 µM, respectively (Fig. 6B), suggesting that the observed growth inhibition is due to off‐target effects. However, the approximately 20‐fold window in IC_50_ for Ri03 against GAS compared to *S. aureus* suggested that growth inhibition could be at least partially dependent on inhibition of dTDP‐L‐rhamnose biosynthesis pathway. To confirm this, we used a biochemical assay in which recombinant RmlB, RmlC and GacA form dTDP‐L‐rhamnose out of the substrate dTDP‐L‐glucose (van der Beek *et al.*, 2015). Ri03 showed a concentration‐dependent inhibition of dTDP‐L‐rhamnose formation with an IC_50_ ~ 166 µM (Fig. 6D). These data suggest that Ri03 directly targets the dTDP‐L‐rhamnose biosynthesis pathway despite having additional off‐target effects *in vivo*.

### Activity of Ri03 against other bacterial species

We extended our experiments to include other human and animal streptococcal species for which dTDP‐L‐rhamnose is an essential nucleotide sugar. Group C *Streptococcus* contains a related surface carbohydrate, the characteristic group C carbohydrate, composed of a polyrhamnose backbone decorated with a di‐GalNAc side chain (Mistou *et al.*, 2016). Ri03 inhibited growth of GCS with an IC_50_ of 0.42 mM (Fig. 6E) and of *S. mutans *with an IC_50_ of 0.41 mM (Fig. 6E). In addition, we also assessed the effect of Ri03 against *M. tuberculosis*, the causative agent of tuberculosis, for which dTDP‐L‐rhamnose biosynthesis is also essential (Ma *et al.*, 2002; Li *et al.*, 2006). Again, Ri03 prevented growth of *M. tuberculosis* at a concentration of 175 µM (Fig. 6F).

## Discussion

In this study, we show that GAS RmlB and RmlC are dTDP‐glucose‐4,6‐dehydratase and dTDP‐4‐keto‐6‐deoxyglucose‐3,5‐epimerase enzymes respectively. Both GAS RmlB and RmlC functionally replaced *S. mutans* homologs in a heterologous expression system. The rescue of growth and morphological phenotypes of the *rmlB* and *rmlC* mutants by plasmid overexpression excludes the occurrence of polar effects within the *rmlACB* operon. In addition to proving the function of both GAS RmlB and RmlC as rhamnose biosynthesis enzymes, we also identified Y159 in RmlB and H76 and K82 in RmlC as critical catalytic residues. Our complementation studies indicate that the mechanism of catalysis is conserved among streptococcal species.

Deletion of *rmlB* and *rmlC* in *S. mutans* resulted in a phenotype similar as previously observed for an *rmlD* deletion mutant (van der Beek *et al.*, 2015), underscoring that inhibition of the dTDP‐rhamnose biosynthesis pathway severely impacts bacterial viability. Indeed, the *rmlABCD* genes are essential for *S. mutans* in the competitive environment of a mutant transposon library (Shields *et al*., 2018), similar to GAS and GCS (Le Breton *et al.*, 2013; 2015; Charbonneau *et al.*, 2017). We are however able to construct *rml* deletion mutants in *S. mutans* in isolation in contrast to similar attempts in GAS (van Sorge *et al.*, 2014). The reason for this discrepancy is currently unclear but may be caused by differential compositions of the cell walls or possibly these deletions are partially rescued through unidentified interacting pathways.

Screening a chemical compound library to identify inhibitors of the dTDP‐L‐rhamnose biosynthesis pathway is methodically challenging. Previously, indirect methods were used to determine the production of the end product dTDP‐L‐rhamnose by monitoring the production of co‐factor NAD(P)H (Graninger *et al.*, 1999; Sivendran *et al.*, 2010; van der Beek *et al.*, 2015). A superior assay would involve a HPLC or mass spectrometry approach where every single step of product formation and substrate consumption could be monitored. However, it is challenging to develop this method for medium or high throughput screening. We, therefore, investigated an approach using recombinant enzymes in their natural state, i.e. with bound co‐factors in their active site. RmlB enzymes require the co‐factors NAD(P), which appears to be tightly bound in the active site as it was co‐purified during RmlB crystallizations (Allard *et al.*, 2002; Beis *et al.*, 2003). This is in agreement with the regeneration of NAD during dTDP‐4‐keto‐6‐deoxy‐D‐glucose synthesis (Allard *et al.*, 2002). GacA/RmlD enzymes also require NADPH as a co‐factor, which appears to be less tightly bound as it was not observed in the GAS GacA and *S. suis* RmlD crystal structures and therefore should be regenerated by a different enzyme (Blankenfeldt *et al.*, 2002; van der Beek *et al.*, 2015). Using the binding approach, we identified seven potential inhibitors that interacted with at least one enzyme of the dTDP‐L‐rhamnose biosynthesis pathway with affinities ranging from 0.1 mM to 80 mM. One of the most potent chemical fragments Ri03, 5‐(4‐chlorophenyl)‐2‐furoic acid, is a small molecule, which binds RmlB and GacA with an affinity of 0.2 and 0.3 mM, respectively, and RmlC with approximately 3 mM. The 2‐furoic acid scaffold of Ri03 is of interest since this organic compound is used as a preservative and flavoring agent in the food industry. The bactericidal and fungicidal activity of 2‐furoic acid is presumably unrelated to rhamnose biosynthesis inhibition, but may be an explanation for the observed rhamnose‐independent inhibition of *S. aureus*. Previous studies have also identified small molecules that inhibit Rml enzymes (Ma *et al.*, 2001; Babaoglu *et al.*, 2003; Sivendran *et al.*, 2010; Wang *et al.*, 2011; Ren *et al.*, 2015). However, none of the predicted or confirmed inhibitors show a structural resemblance to Ri03.

Of particular interest is the observation that Ri03 also inhibits growth of *M. tuberculosis*. As drug‐resistant tuberculosis continues to be a public health crisis and specifically multi‐ and extensively drug‐resistant tuberculosis cure rates are below 60 and 30%, new chemical entities with activity against *M. tuberculosis* are urgently required. Interestingly, Ri03 has a similar chemical scaffold as another anti‐tuberculosis compound that was recently reported, which has an MIC_50_ of 135 µM (Negatu *et al.*, 2018; 5‐(4‐Chlorophenyl)‐N,N,2‐trimethyl‐3‐furamide, compound F8). It is unknown whether F8 targets the dTDP‐L‐rhamnose biosynthesis pathway, but this is certainly something that should be investigated. If F8 also was inhibiting the dTDP‐rhamnose biosynthesis pathway, these lead structures together with available structural and biochemical information on enzymes of the rhamnose biosynthesis pathway, should guide future attempts for rational drug design to optimize specificity as well as solubility of Ri03 and F8.

Fragments such as the ones that we identified here, are generally known to have low affinity and limited specificity. Indeed, using a bacterial species which does not synthesize rhamnose (*S. aureus*), we observed that Ri03 has off‐target effects that attenuated bacterial growth. Nonetheless, Ri03 is approximately 10‐ to 20‐fold more effective at inhibiting growth of species for which rhamnose is essential, i.e. three streptococcal species as well as *M. tuberculosis*. The high sequence identity of RmlB, RmlC and GacA/RmlD among GAS, GCS and *S. mutans *(92–94%, 83% and 82–87% protein sequence identity respectively; Fig. 2C) supports the observation that Ri03 shows similar IC_50_ values against all three strains. For *M. tuberculosis*, protein sequence identity is less apparent and highest for RmlB (61%, 30% and 35% for RmlB, RmlC and GacA respectively). This may indicate that the observed mycobacterial growth inhibition of Ri03 predominantly occurs through interference with RmlB function, which coincides with the fact that Ri03 has the highest affinity for this rhamnose biosynthesis enzyme. Complementary, we have proven that Ri03 acts specifically on the dTDP‐L‐rhamnose biosynthesis pathway using a biochemical assay.

Future experiments aim to investigate the binding mode of Ri03 on a structural level to the proteins RmlB, RmlC and GacA to elucidate whether Ri03 inhibits RmlB, RmlC and GacA by competing with the nucleotide‐sugar and if a common binding mode is observed among the different enzymes. With more functional and structural knowledge, Ri03 can be further optimized to identify more potent and specific derivatives with greater potency targeting the GAS and related homologous Rml enzymes in other pathogenic species.

## Experimental procedures

### Bacterial strains and growth conditions

Bacterial strains used in this study are described in Table 2. *S. mutans *Xc (Koga *et al.*, 1989), a serotype c wild‐type (WT) strain, was a kind gift of Dr. Y. Yamashita (Kyushu University, Japan) and was routinely grown in Todd‐Hewitt Broth (THB, Oxoid) or on THB agar at 37°C with 5% CO_2_. When appropriate, *S. mutans *was cultured with 10 µg ml^−1^ erythromycin (ERY) or 3 µg/ml chloramphenicol (CHL). GAS 5448, a representative of the epidemic M1T1 clone, was cultured in Todd‐Hewitt Broth (Becton Dickinson) supplemented with 1% yeast extract (THY; Oxoid) or on THY agar at 37°C. *E. coli *MC1061 was used for cloning purposes and was grown in lysogeny broth (LB, Oxoid) or on LB agar containing 10 µg/ml CHL at 37°C. *Mycobacterium tuberculosis* laboratory strain H37Rv was routinely cultured in Middlebrook 7H9 broth (Difco) supplemented with 10% (v/v) Albumin Dextrose Catalase (ADC) enrichment (Difco), 0.05% (v/v) tyloxapol and 0.02% (v/v) glycerol. Liquid cultures were grown at 37°C in 50 mL centrifugation tubes with rotation of 40 rpm until mid‐exponential phase (OD_600_ ~ 1). For solid growth drug susceptibility experiments, *M. tuberculosis* H37Rv was grown in Middlebrook 7H10 (Difco) agar medium supplemented with 10% (v/v) of Oleic acid ADC enrichment (OADC) (Difco) and 0.5% (v/v) glycerol (c7H10) at 37°C incubator.

**Table 2:** Bacterial strains used in this study

Bacterial strains	Abbreviation	Resistance
*S. mutans* Xc wild type (serotype c)	SMU WT	–
*S. mutans* Xc *ΔrmlB*	SMU Δ*rmlB*	ERY
*S. mutans* Xc *ΔrmlB* + pDC123_SMU_*rmlB*	SMU Δ*rmlB* + pSB	ERY + CHL
*S. mutans* Xc *ΔrmlB* + pDC123_GAS_*rmlB*	SMU Δ*rmlB* + pGB	ERY + CHL
*S. mutans* Xc *ΔrmlB* + pDC123_GAS_*rmlB*_Y159F	SMU Δ*rmlB* + pGB_Y159F	ERY + CHL
*S. mutans* Xc *ΔrmlC*	SMU Δ*rmlC*	ERY
*S. mutans* Xc *ΔrmlC* + pDC123_SMU_*rmlC*	SMU Δ*rmlC* + pSC	ERY + CHL
*S. mutans* Xc *ΔrmlC* + pDC123_GAS_*rmlC*	SMU Δ*rmlC* + pGC	ERY + CHL
*S. mutans* Xc *ΔrmlC* + pDC123_GAS_*rmlC*_H76N	SMU Δ*rmlC* + pGC_H76N	ERY + CHL
*S. mutans* Xc *ΔrmlC* + pDC123_GAS_*rmlC*_K82A	SMU Δ*rmlC* + pGC_K82A	ERY + CHL
*S. pyogenes *5448 wild type (serotype M1T1)	GAS	–
*S. equi *subsp. *zooepidemicus *MGCS10565	GCS	–
*E. coli *MC1061	–	–
*E. coli *BL21 (DE3)	–	–
*E. coli *DH5alpha	–	–
*Staphylococcus aureus* *NRS384*	–	MRSA
*M. tuberculosis *H37Rv	–	–

### Cloning, expression and purification of GAS enzymes and biochemical assay

GAS RmlB, RmlC and GacA proteins were produced and purified as described previously (van der Beek *et al.*, 2015). The IC_50_ of Ri03 against the dTDP‐rhamnose biosynthesis pathway enzymes RmlB, RmlC and GacA was determined using the published procedure (van der Beek *et al.*, 2015). In short, the assay buffer system contained 25 mM Tris‐base (pH 7.5), 150 mM NaCl, 0.2 mM NADPH and 5 pM of recombinant GAS RmlB, RmlC and GacA. The assay was started with the addition of 400 µM dTDP‐D‐glucose. The oxidation of NADPH to NADP+ was measured by the change in intrinsic absorbance at 340 nm using a SpectraMax M2 plate reader. Background absorbance was subtracted and IC_50_ value was determined using the nonlinear fit model in GraphPad Prism.

### Genetic manipulation of *S. mutans*

*Streptococcus mutans *is naturally competent and was transformed as described previously. Shortly, bacteria were grown in THB containing 5% heat‐inactivated horse serum (BioRad) and supplied with 500 ng knockout construct or complementation plasmid (van der Beek *et al.*, 2015). Cultures were plated on THB agar plates containing the appropriate antibiotics. Single colonies were selected and verified for the deletion of *rmlB*/*rmlC* and/or the presence of complementation plasmid using colony PCR and sequencing.

Deletion mutants of *rmlB* (SMU Δ*rmlB*) and *rmlC* (SMU Δ*rmlC*) were obtained via the addition of a knockout construct to competent *S. mutans* WT. This construct consisted of an ERY resistance cassette with ~700 bp flanking regions homologous to the up‐ and downstream regions of *rmlB* or *rmlC*. A detailed cloning strategy is available in the supplemental material and Table S1.

### *S. mutans* complementation plasmids

*S. mutans rmlB/rmlC* and GAS *rmlB/rmlC *were amplified from gDNA using primers containing XbaI, XhoI or BamHI restriction site (Table S1). Digested PCR products were subsequently ligated into expression plasmid pDC123 and propagated in *E. coli* MC1061 to obtain large quantities of complementation plasmid. Point mutations were introduced by mutagenesis PCR, using overlapping primers with the corresponding mutation site integrated. The constructed plasmids with the single point mutations were all confirmed by DNA sequencing.

SMU Δ*rmlB* and SMU Δ*rmlC* knockout mutants were not transformable likely resulting from the severe growth defects. Therefore, SMU Δ*rmlB* and SMU Δ*rmlC* strains complemented with *S. mutans rmlB/rmlC* or GAS *rmlB/rmlC* were constructed using a two‐step approach. First, *S. mutans *WT was transformed with the complementation plasmid as described above. Next, these complemented strains were transformed with the respective *rmlB *or *rmlC* knockout constructs and selected for double antibiotic resistance.

### Scanning Electron Microscopy

Scanning electron microscopy was performed as described previously by van der Beek *et al*. (2015). In short, bacteria were grown to mid‐exponential phase, except when incubated with rhamnose inhibitor. In this case, bacteria were cultured as described below in bacterial growth inhibition assays and grown overnight. Bacteria were subsequently washed, fixed, dehydrated, mounted onto 12.5 mm specimen stubs (Agar scientific, Stansted, Essex, UK) and coated with gold to 1 nm using a Quorum Q150R S sputter coater at 20 mA. Samples were visualized with a Phenom PRO desktop scanning electron microscope (Phenom‐World BV) at an acceleration voltage of 10 kV.

### *S. mutans* growth curves

Overnight cultures of *S. mutans *strains with an optical density at 600 nm (OD_600_) higher than 0.35 were diluted 10 times and grown for 1.5 h to early exponential phase. Cultures were then diluted to OD_600_ 0.03 and OD_600_ was measured manually every 30 min. For cultures with an overnight OD_600_ below 0.35, the initial 10‐fold dilution and growth step was omitted. Instead, such overnight cultures were directly diluted to OD_600_ 0.03 and OD_600_ was measured every hour. All cultures were incubated at 37°C with 5% CO_2_ in between measurements.

### Cell wall carbohydrate composition analysis

Cell wall polysaccharides were isolated from *S. mutans,* hydrolyzed and analyzed by chromatography as described previously by van der Beek *et al*. (2015). In short, bacterial cells were harvested from 2 to 5 L cultures and disrupted in 0.1 M citrate buffer (pH 4.6) using a bead beater (Biospec). Cell walls were collected by centrifugation and boiled for 1 h at 100°C in 0.1 M sodium acetate (pH 4.6) containing 4% sodium dodecyl sulfate. Samples were subsequently treated with RNase, DNase, pronase E and trypsin. Cell walls containing peptidoglycan and the serotype C carbohydrate were lyophilized before hydrolysis with TFA. Finally, carbohydrate analysis of monosaccharides was performed on a Dionex ICS‐3000 Ion Chromatography System (Dionex/Thermo Scientific) using a CarboPac PA20 (Dionex/Thermo Scientific) 3 × 150 mm column, equipped with a ICS‐3000 Electrochemical Detector (Dionex/Thermo Scientific). Monosaccharides from all samples were eluted from the column with 12% 0.2 M NaOH, except for monosaccharides from SMU Δ*rmlB* and Δ*rmlC*, which were eluted with 8% 0.2 M NaOH. The ‘Carbohydrates (Standard Quad)’ waveform was used for detection.

### BLI screen using recombinant RmlB, RmlC and GacA

The Maybridge library of fragment‐like molecules (Ro3) was purchased from Maybridge (USA). All three GAS proteins, RmlB, RmlC and GacA, were screened against the library using identical protocols. Proteins were biotinylated on primary amine amino acid side chains using the Thermo Scientific^TM^ EZ‐Link^TM^ NHS‐PEG_4_‐Biotin reagent. The enzymes were incubated with NHS‐PEG_4_‐Biotin in a 1:1 molar ratio at 150 µM for 45 min at room temperature. The reaction buffer contained 25 mM HEPES, pH 7.5, 150 mM NaCl and 0.02 mM TCEP. Excess NHS‐PEG_4_‐biotin reagent was removed using 2 mL Zeba desalting spin columns (Thermo Scientific). Successful biotinylation on primary amines was investigated via Western blotting and probing with ExtrAvidin®−Peroxidase antibody, 1:10,000 (Sigma E2886, Fig. S1A). Before compound screening, the proteins were loaded onto a parallel set of superstreptavidin biosensors (SSA) by incubation in buffer for 900 s. Protein concentrations suitable for the experiments were determined in a series of dilutions. Final screening was conducted at concentrations of: 50 µg/mL RmlB, 25 µg/mL RmlC and 12.5 µg/mL GacA. All compounds were at 2% DMSO concentration. The sensor was blocked by immersion in biocytin (10 µg/mL) for 30 s.

Initial library screening was performed at a compound concentration of 200 µM. Hits were identified by plotting the response rate of every single compound after background subtraction. In total, 12 compounds were validated in 6‐point concentration series using threefold dilutions, ranging from 670 µM to 3 µM. Data were processed and kinetic parameters were calculate using the ForteBio software. Binding curves were manually inspected and approximate binding constants K_D_ are listed in Table S3. Fragments that did not induce concentration‐dependent signal changes, i.e. did not bind to the recombinant enzymes, are not displayed in Fig. S2.

### Bactericidal activity

GAS 5448, GCS MGCS10565 and *S. mutans *Xc strains (Table 2) were grown in appropriate bacterial culture broth overnight at 37°C. Bacterial cultures were diluted 10 times and grown to mid‐log phase (OD_600_ 0.4). Cultures were diluted 200 times in culture medium followed by a twofold dilution with various concentrations of rhamnose inhibitor or DMSO. OD_600_ was recorded every 15 min at 37°C with 5 sec medium shaking before measurement for GAS, GCS and *S. mutans* using either a Bioscreen C MBR machine (Growth Curves AB Ltd, Oy, Finland) or a Synergy 2 plate reader (Biotek). OD_600_ values were plotted against the concentration of compound after 9 h of growth using GraphPad Prism 7. IC_50_ values were calculated using a nonlinear four‐parameter dose–response curve with variable slope. Because high concentrations were not always feasible due to solubility issues, a high concentration of compound (50 mM) was artificially introduced where appropriate and set to zero to obtain more reliable dose–response curves.

### Minimum inhibitory concentration assay in *M. tuberculosis*

To determine the effect of inhibitors of rhamnose biosynthesis against *M. tuberculosis*, we performed an agar‐based spot culture growth inhibition assay (Evangelopoulos and Bhakta, 2010). Briefly, compound Ri03 was dissolved in DMSO at 20 mM concentration and serial dilutions (800 µM to 12.5 µM) was added in each well of a 24‐well plate, followed by the addition of 2 mL of warm (55°C) c7H10 agar media. Once solid, the plates were inoculated with a diluted culture of *M. tuberculosis* H37Rv so that a spot (2 µL) of around 1000 CFUs was placed in the middle of each well. The plates were incubated at 37°C incubator for 14 days and the MIC_99_ was determined as the well with the lowest concentration of Ri03 where there was no bacteria growth. Rifampicin (a first line drugs against tuberculosis) was also used in parallel as control. The concentration of DMSO (0.1% v/v) remained constant in all wells, including the no drug control wells. Two independent experiments were performed for MIC determination.

## Author contributions

Conception and design of the study: SvdB, AZ, HCD, NvS; Acquisition, analysis and interpretation of the data: SvdB, AZ, EC, RNC, BHM, DE, LPSC, GJB, HCD, NvS; Writing of the manuscript: SvdB, AZ, HCD, NvS.
