# Supplementary figures and images for: Streptococcal dTDP‐L‐rhamnose biosynthesis enzymes: functional characterization and lead compound identification
Source: Mol Microbiol. 2019 Jan 31;111(4):951–64. doi: 10.1111/mmi.14197 (PMC6487966; doi:10.1111/mmi.14197)

# Figure S1

**A**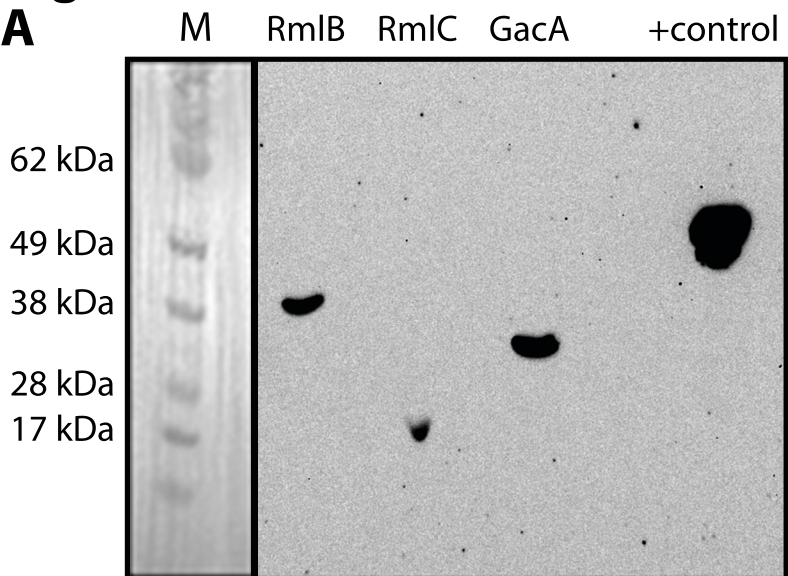**B**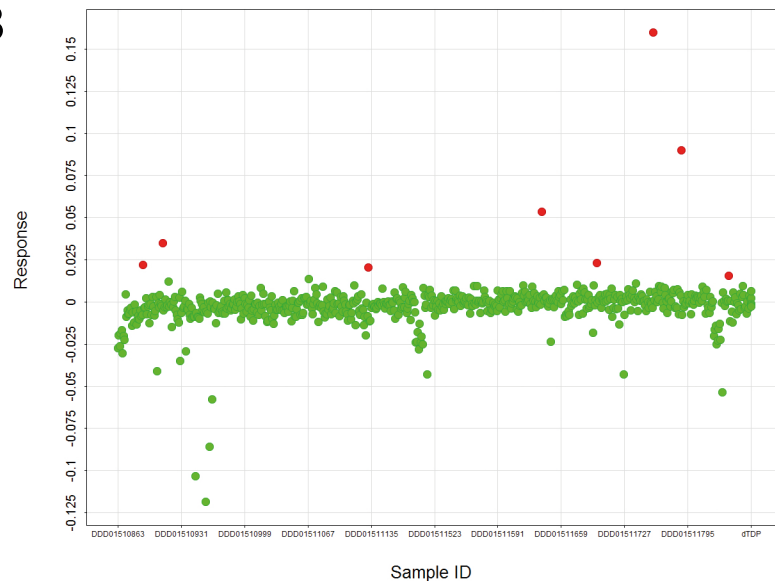**C**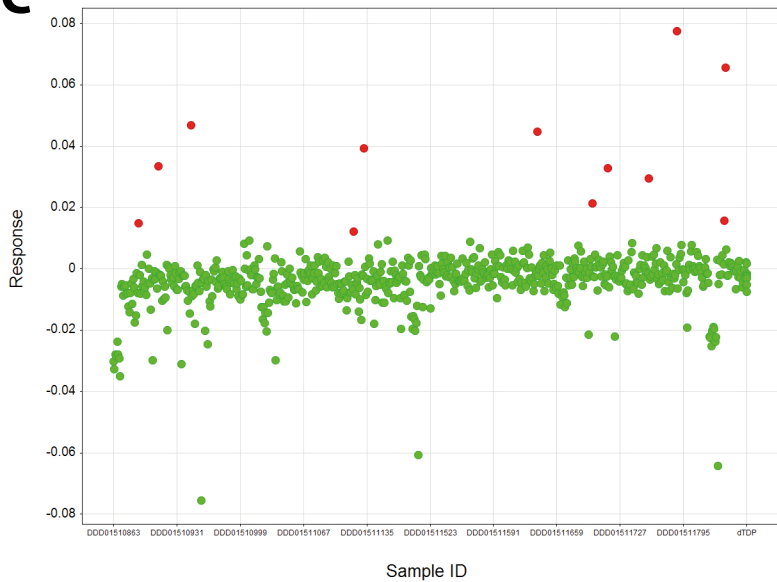**D**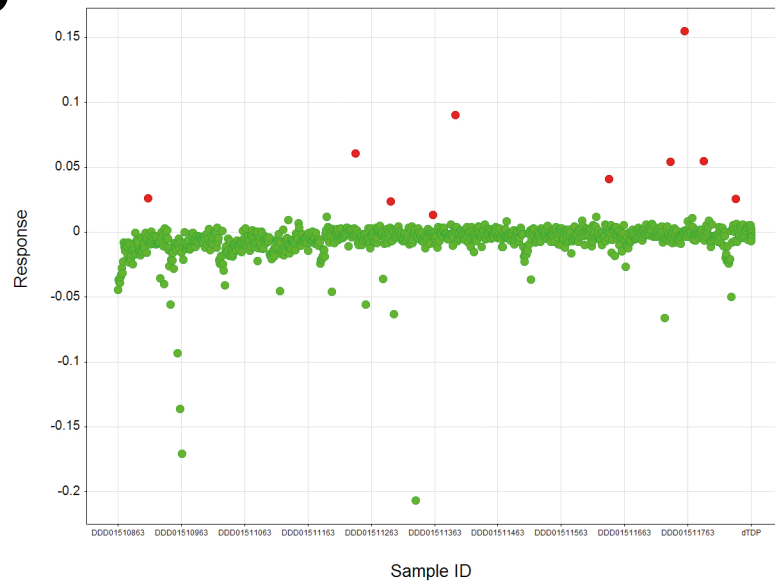

Supplement: Supplementary file 1 [file MMI-111-951-s001.pdf]

# Figure S2

Ri01

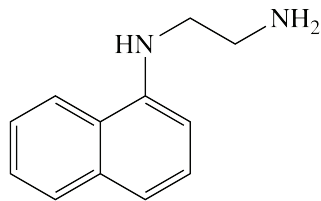

Ri02

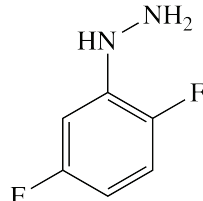

Ri03

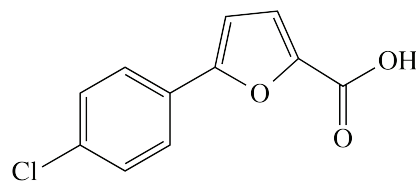

Ri04

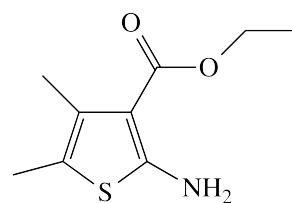

Ri06

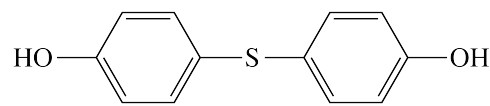

Ri07

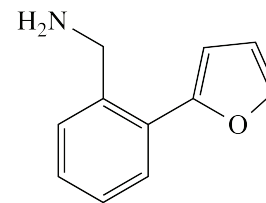

Ri08

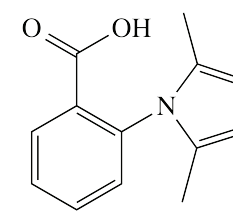

*SpRmlB*

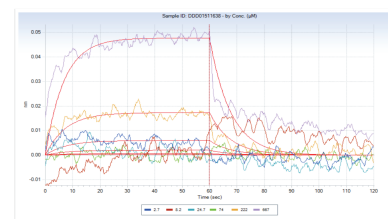

$K_D \sim 4.8$

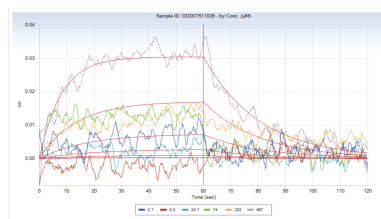

0.4

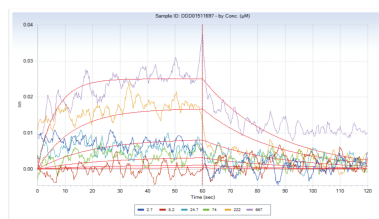

0.2

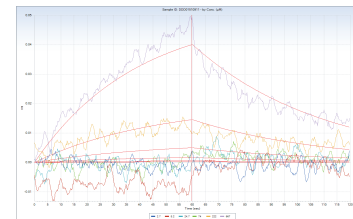

30

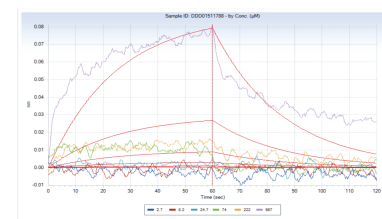

17

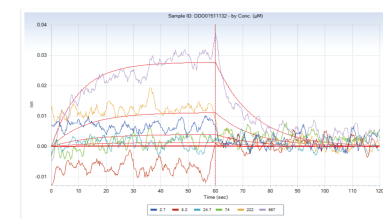

1.8

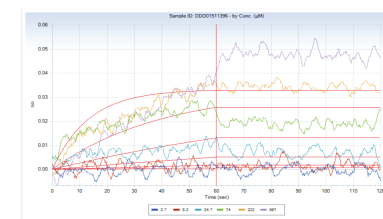

>0.1

*SpRmlC*

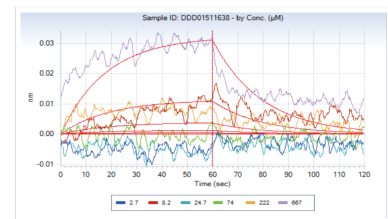

$K_D \sim 7.8$

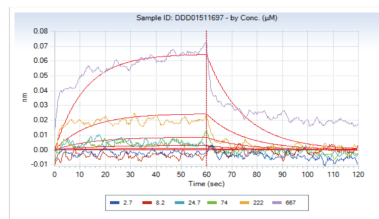

3.0

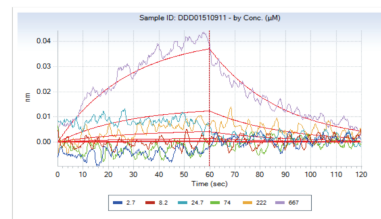

80

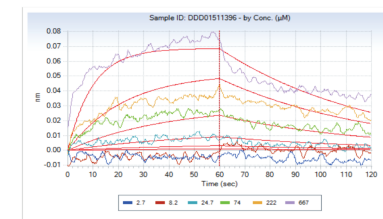

0.1

*SpGacA*

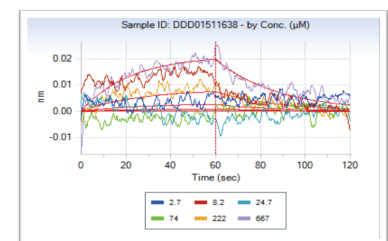

$K_D \sim 2.5$

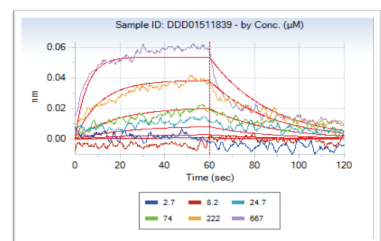

0.2

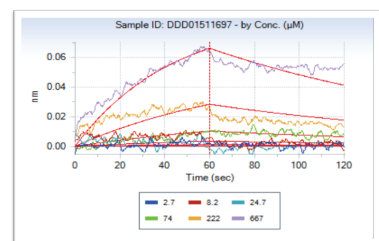

0.3

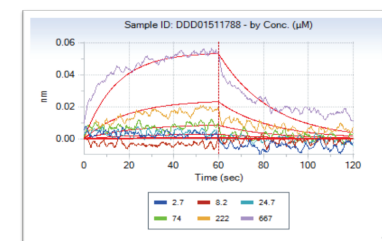

1.1

Supplement: Supplementary file 2 [file MMI-111-951-s002.pdf]
