# Supplementary material for: Streptococcal dTDP‐L‐rhamnose biosynthesis enzymes: functional characterization and lead compound identification
Source: Mol Microbiol. 2019 Jan 31;111(4):951–64. doi: 10.1111/mmi.14197 (PMC6487966; doi:10.1111/mmi.14197)
Supplement: Supplementary file 3 [file MMI-111-951-s003.pdf]

Supplementary information

Streptococcal dTDP-L-rhamnose biosynthesis enzymes: functional characterization and lead compound identification

Samantha L. van der Beek[a], Azul Zorzoli[b], Ebru Çanak[a], Robert N. Chapman[c], Kieron Lucas[b], Benjamin H. Meyer[b], Dimitrios Evangelopoulos[d], Luiz Pedro S. de Carvalho[d], Geert-Jan Boons[c,e], Helge C. Dorfmueller[b,]# and Nina M. van Sorge[a,]#

1. University Medical Center Utrecht, Utrecht University, Medical Microbiology, Heidelberglaan 100, 3584 CX, Utrecht, The Netherlands.
2. Division of Molecular Microbiology, University of Dundee, School of Life Sciences, Dow Street, DD1 5EH, Dundee, UK.
3. Complex Carbohydrate Research Center, Department of Chemistry, The University of Georgia, 315 Riverbend Road, Athens, USA
4. Mycobacterial Metabolism and Antibiotic Research Laboratory, The Francis Crick Institute, London, UK
5. University Utrecht, Utrecht Institute Pharmaceutical Science, Department of Medical Chemistry and Chemical Biology, 3508 TB, Utrecht, The Netherlands

# Address correspondence to NM van Sorge, nsorge3@umcutrecht.nl and HC Dorfmueller, h.c.z.dorfmueller@dundee.ac.uk

Running Head: Function and inhibition of dTDP-L-rhamnose enzymes

Method S1 Cloning strategy of knock-out constructs and complementation plasmids for *S. mutans.*

Knock-out constructs for the deletion of *rmlB* and *rmlC* were made in several steps. First, upstream regions of *rmlB* and *rmlC* were amplified from *S. mutans* gDNA using a forward primer ~700 bp upstream of *rmlB* (P1) or *rmlC* (P13) and a reverse primer directly upstream of *rmlB* (P2) or *rmlC* (P14) with an ERY overhang of 30 bp at the 5'-end. Similarly, downstream regions were amplified using reverse primers ~700 bp downstream of *rmlB* (P4) or *rmlC* (P16) and forward primers directly downstream of *rmlB* (P3) or *rmlC* (P15) with an ERY overhang of 30 bp attached to the 5'-end. The ERY resistance cassette was amplified from plasmid pDCerm (P5 + P6). The upstream and downstream regions were subsequently ligated to the ERY resistance cassette using a single pcr step to create the Δ*rmlB* and Δ*rmlC* constructs used for transformation.

Fig. S1 Maybridge library screening data.

(A) Anti-biotin Western blot of purified RmlB, RmlC and GacA proteins (RmlB = 39 kDa, RmlC = 23 kDa, GacA = 32 kDa), with positive control (biotinylated Protein X; 55 kDa). (B) RmlB, (C) RmlC and (D) GacA hit cut-off value figures from BLI screen. The binding curve of all compounds were analyzed with robust calculations and plotted as compound (X-axis) vs. response. Potential hits above the hit cut-off are depicted in red. In total 12 compounds were selected and tested in dose-dependent BLI studies against all three target enzymes.

Fig. S2 Binding studies of rhamnose inhibitors to RmlB, RmlC and GacA.

Chemical structures of identified inhibitors, binding curves vs. purified recombinant proteins and calculated binding affinities (K_D) in mM.

Table S1. Primer used for cloning of *S. mutans* (SMU) *rmlB* and *rmlC* mutants

Primer	Origin	Sequence	Template
**Upstream ΔrmlB construct + ERY overhang**			
**P1**	rmlB up Fwd	AGCTGATAAAATCAATCGCAAAGAC	SMU Xc
**P2**	rmlB up Rev + **ERY**	**GTTTTGAGAATATTTTATATTTTTGTTCAT**ATATTAAGATACAAAGGGG CGATTCA	SMU Xc
**Downstream ΔrmlB construct + ERY overhang**			
**P3**	rmlB down Fwd + **ERY**	**AGTTATCTATTATTTAACGGGAGGAAATAA**AGATAAATAAGAGGCTG GGACAAAAGT	SMU Xc
**P4**	rmlB down Rev	CAATAAAAGCTCGCACCCGTT	SMU Xc
**Erythromycin resistance cassette**			
**P5**	ERY Fwd	ATGAACAAAAATATAAAATATTCTCAAAACTTTTTAACG	pDCerm
**P6**	ERY Rev	TTATTTCCTCCCGTTAAATAATAGATAACT	pDCerm
**pDC123_SMU_rmlB**			
**P7**	SMU_rmlB.XbaI Fwd	GCTCTAGAATGACAGAATATAAAAACATTATCGTTACCG	SMU Xc
**P8**	SMU_rmlB.BamHI Rev	CGCGGATCCTTAATTAAGTATTTTTTGTGTTTTGGCATAG	SMU Xc
**pDC123_GAS_rmlB(_Y159F)**			
**P9**	GAS_rmlB.XbaI Fwd	GCTCTAGAATGTATAAAAATATTATCGTAACTGGTGGAGC	GAS 5448
**P10**	GAS_rmlB.BamHI Rev	CGCGGATCCTTATTTAATCACTTCTTGAGTTTTAGCATACTTG	GAS 5448
**pDC123_GAS_rmlB_Y159F**			
**P11**	GAS_rmlB. Y159F Fwd	CATCATCACCTTTCTCATCAACTAAGG	GAS 5448
**P12**	GAS_rmlB. Y159F Rev	CCTTAGTTGATGAGAAAGGTGATGATG	GAS 5448
**Upstream ΔrmlC construct + ERY overhang**			
**P13**	rmlC up Fwd	AGCCAGAACACCCTAAATCACACTA	SMU Xc
**P14**	rmlC up Rev + **ERY**	**GTTTTGAGAATATTTTATATTTTTGTTCAT**TTTAAGCTTCTCCAATCAAA CGGA	SMU Xc
**Downstream ΔrmlC construct + ERY overhang**			
**P15**	rmlC down Fwd + **ERY**	**AGTTATCTATTATTTAACGGGAGGAAATAA**TGTTTGAAATCTTCTTAG AGATTGCAA	SMU Xc
**P16**	rmlC down Rev	TCGCCTTATTTCTCTAGTACTTTTTTCAG	SMU Xc
**pDC123_SMU_rmlC**			
**P17**	SMU_rmlC.XbaI Fwd	GCTCTAGAATGAACAAAAATATAAAATATTCTCAAAACTTTTTAACG	SMU Xc
**P18**	SMU_rmlC.BamHI Rev	CGCGGATCCTCACAAATCTTCTGCTTTTAATGGTTT	SMU Xc
**pDC123_GAS_rmlC**			
**P19**	GAS_rmlC.XbaI Fwd	GCTCTAGAATGACAGAAACTTTTTTTGACAAACCATT	GAS 5448
**P20**	GAS_rmlC.BamHI Rev	CGCGGATCCCTATAGGTCTTTTGGTTTCAGTGGTTTG	GAS 5448
**pDC123_GAS_rmlC_H76N**			
**P21**	GAS_rmlC. H76N Fwd	CCAAGGTTCAGCGTTAAGTCCACGCAGCAC	GAS 5448
**P22**	GAS_rmlC. H76N Rev	GTGCTGCGTGGACTTAACGCTGAACCTTGG	GAS 5448
**pDC123_GAS_rmlC_K82A**			
**P23**	GAS_rmlC. K82A Fwd	CAACTGAGATGTAAGCATCCCAAGGTTCAG	GAS 5448
**P24**	GAS_rmlC. K82A Rev	CTGAACCTTGGGATGCTTACATCTCAGTTG	GAS 5448
**pDC123_GAS_rmlC_H76N/K82A**			
**P25**	GAS_rmlC.XhoI Fwd	CAAGCTCGAGGAATGTCTTCACAACGC	GAS 5448
**P26**	GAS_rmlC. XbaI Rev	CGCGGAGCTCTAGAATGACAGAAAC	GAS 5448

Table S2. **Protein accession numbers for RmlB, RmlC and GacA homologs used for sequence alignments**

	Protein accession number	
**Species**	**RmlB**	**RmlC**
Streptococcus pyogenes	WP_011285494.1	WP_012560640.1
Salmonella enterica	WP_023226833.1	WP_038395428.1
Streptococcus agalactiae	WP_016502954.1	WP_017769218.1
Streptococcus anginosus	PLA73052.1	WP_049516748.1
Streptococcus dysgalactiae	WP_065361279.1	WP_003057688.1
Streptococcus equi	WP_014623057	WP_041790386.1
Streptococcus equinus	ARC34160.1	ARC34159.1
Streptococcus mutans	WP_002273831.1	WP_002283091
Streptococcus pneumoniae	NP_357916.1	WP_000131446
Streptococcus suis	WP_044759933.1	BAM95164.1
